# A Feasibility Trial on Intranasal Evaporative Cooling for Acute Migraine in an At-Home Setting

**DOI:** 10.7759/cureus.72911

**Published:** 2024-11-03

**Authors:** Moa Wolff, Ingunn Winnberg, Erling Tronvik, Mohammad F Bakhsheshi, Patrik Midlov

**Affiliations:** 1 Department of Clinical Sciences, Lund University, Malmö, SWE; 2 Department of Neuromedicine and Movement Science, St. Olavs University Hospital, Trondheim, NOR; 3 Medicon Village, BrainCool AB, Lund, SWE

**Keywords:** at-home, evaporative cooling, feasibility trial, intranasal administration, migraine, pilot study

## Abstract

Background: A significant proportion of people with migraine do not achieve sufficient relief of their acute migraine symptoms with the currently available medications. A previous study showed that intranasal evaporative cooling reduced headache and migraine-associated symptoms when given in an outpatient clinic setting. This study aimed to evaluate the feasibility of self-administering the same intervention for acute migraine in an at-home setting. The findings of this study were intended to inform the design and implementation of a planned full-scale randomized controlled trial (RCT).

Methods: We conducted a prospective single-group clinical feasibility trial in southern Sweden. Participants meeting the criteria for episodic migraine, with or without aura, were recruited through local advertisements. After a screening period, during which two migraine attacks were registered and evaluated under usual care, participants treated their next three migraine attacks at home with 10 minutes of intranasal cooling (RhinoChill®, BrainCool AB, Lund, Sweden). The primary outcome was a reduction in headache, nausea, photophobia, and phonophobia immediately after treatment. The secondary outcome was tolerability, and treatment effects within 24 hours.

Results: Six out of 15 participants completed the study, using the cooling treatment for three consecutive migraine attacks. The main reasons for drop-out were pain/discomfort from treatment and lack of effect. A total of 23 treatments were registered by 10 participants. Small effects on pain and other migraine symptoms were observed immediately after treatment. The treatment was considered very unpleasant (Visual Analogue Scale 7.3/10) and not superior to usual care.

Conclusions: The RhinoChill® intranasal cooling treatment at home was found to be non-feasible due to pain and discomfort, resulting in a high drop-out rate. Additionally, it had only minor effects on migraine pain and symptoms. The findings of this study led to the cancellation of a planned full-scale RCT.

## Introduction

Migraine is ranked as the second most disabling disorder globally according to the Global Burden of Disease (GBD) study 2019 [1]. The climb from the previous ranking (sixth most disabling disorder, GBD 2016) is partly due to that 70% of medication overuse headache burden has now been reattributed to migraine [2]. The recent ranking highlights two major concerns, the first being that migraine has considerable economic consequences for the patient and for society [3], and the second being that acute migraine treatment in itself may cause a vicious circle with medication overuse and increased disability [4]. Hence, the need to find new solutions to alleviate migraine symptoms and its consequences is significant.

Scientific documentation about cooling treatments as a means for treating headaches dates back to at least 1849 [5]. In the modern era, applying cooling treatments is the most common self-administered pain-relieving maneuver in persons with migraine without aura, and the second most common maneuver (after compression) in persons with migraine with aura [6]. Several cold interventions (e.g., cold-gel headband, cold-gel cap, intraoral cooling), have proven to be effective in acute migraine pain reduction [7-11].

Migraine is a neurovascular disorder linked to trigeminal nerve activation and sensitization [12]. Nociceptive nerve fibers from the trigeminal ganglion innervate meningeal and large cerebral arteries. Activation of these structures results in cranial vasodilatation mediated by the release of vasoactive neuropeptides including the calcitonin gene-related peptide (CGRP) 2 [13].

The proposed mechanisms of how cold interventions relieve migraine symptoms include that cold induces vasoconstriction with decreasing blood flow and that cooling induces analgesia through decreased velocity of nerve conduction [10]. It is also possible that cold therapy has a more direct effect on the trigeminovascular pain signaling system through effects on temperature-sensitive transient receptor potential (TRP) channels found in trigeminal ganglion [14]. TRPM8 (Transient Receptor Potential Melastatin 8), which mediates cold sensation, has been linked to migraine pathophysiology in genome-wide association studies (GWAS) [15].

In 2015 a prospective observational pilot study was performed in Great Britain to investigate the effect of the RhinoChill® (BrainCool AB, Lund, Sweden) intranasal cooling system for the acute relief of migraine in an adult population [16]. The pilot study was performed in a hospital out-patient clinic and showed a statistically significant reduction of pain and associated symptoms of migraine at five and 10 minutes (during treatment) and at one and two hours following treatment along with a significant effect on pain and migraine-associated symptoms at 24 hours (all p values <0.001). The results were based on 20 administered treatments to 15 participants.

If intranasal cooling is to be relevant as a treatment for a larger migraine population, it needs to be self-administered by the patient at home.

The aim of this subsequent pilot study was to evaluate the feasibility, effect, and tolerability of self-administered RhinoChill® intranasal cooling on migraine headaches and migraine-associated symptoms in an at-home setting.

The primary objective of the trial was as follows: (1) to assess treatment effect on migraine symptoms directly after treatment.

The secondary objectives of the trial were as follows: (1) to assess the tolerability of treatment and any side effects/adverse events, and (2) to assess the effect on migraine symptoms at one, two, and 24 hours after treatment.

The results from this pilot study were intended to further develop and improve the intervention and study design prior to the implementation of a large-scale multicenter randomized control trial (RCT) in Sweden and Norway. The pilot study was initiated by the sponsor, BrainCool AB.

## Materials and methods

Trial design

We conducted a single-group clinical pilot trial, evaluating the effect and tolerability of self-administered RhinoChill® intranasal cooling for the acute treatment of migraine headaches and migraine-associated symptoms in an at-home setting.

Important changes to methods after trial commencement

Due to slow recruitment in autumn 2020, the recruitment strategy was changed from using the Lund University (LU) website and LU Facebook to also including advertisements in the local newspaper. In autumn 2020, a new inclusion criterion was also introduced: "Living in the Malmö-Lund area", since many of those who expressed interest in participating in the study lived too far from Lund for participation to be possible.

Setting, participants, and recruitment

The study was conducted in 2021 in Lund, a city in Sweden’s southernmost region. Lund has approximately 92,000 inhabitants, and LU students make up about a third of the city’s population.

Participants were recruited through an advertisement in the local city paper, on the LU website, and on LU Facebook. People who were interested in participating were able to register through e-mail or telephone. They were then contacted by a researcher/physician by telephone for a brief eligibility check and to book a first meeting at the BrainCool office in Lund. At the baseline visit, performed by the same researcher/physician who conducted the telephone interview, eligibility criteria were once more proved via an interview (see inclusion and exclusion criteria below) and anterior rhinoscopy was conducted to exclude intranasal obstruction. The participants also received a demonstration of the RhinoChill® cooling device and were able to test the insertion of the silicon nose-canula through which the evaporative coolant is administered. If the participant remained interested in participating in the study after the information, informed consent was obtained.

The study was approved by the Regional Ethical Review Board in Lund, Sweden (Dnr: 2019/05471) 2020-05-08, and was registered at Clinicaltrials.gov (NCT04660864), https://clinicaltrials.gov/study/NCT04660864.

Inclusion Criteria

Of age 18-70 years, meeting the International Classification of Headache Disorders (2nd edition) [17]criteria for episodic migraine, with or without aura, migraine diagnosis >1 year, migraine attacks two to eight times/month, living in Malmö-Lund area and, reliable contraception (fertile women).

The inclusion criterion of at least two migraine attacks per month was set to avoid prolonged screening and intervention periods. The upper limit of eight attacks per month was established to exclude individuals with chronic migraine.

Exclusion Criteria

Any change of migraine prophylaxis within three months prior to the study commencing, failure of participant to adhere to protocol requirement, smoker or smoker in participant’s household, prior nose surgery or intranasal obstruction, pregnancy, breastfeeding, or planned pregnancy during the trial period, oxygen dependency, medical history of skull base fracture or severe facial trauma or, no migraine attacks during prolonged screening phase (60 days).

During a screening period of one month with treatment as usual, participants were instructed to record their migraine symptoms, any given treatment, and treatment effects. After a minimum of two migraine attacks, the participants received individual instructions on how to use the RhinoChill® system during a home visit from the BrainCool support team.

During the following treatment period, participants were instructed to treat their upcoming three migraine attacks with 10 minutes of nasal cavity cooling and register symptoms at 10 minutes (directly after treatment), one hour, two hours, and 24 hours, respectively. Symptom scores were registered on paper forms that were sent through postal mail to the researchers. The participants were asked to avoid rescue medication within the first two hours after cooling treatment to enable them to distinguish between cooling and medicine treatment effects.

After three treatments a new home visit was booked, where the BrainCool support team retrieved the device and registered any side effects/adverse events. Participants could provide feedback on the intervention using free-text sections on the paper forms.

Intervention

**Figure 1:**
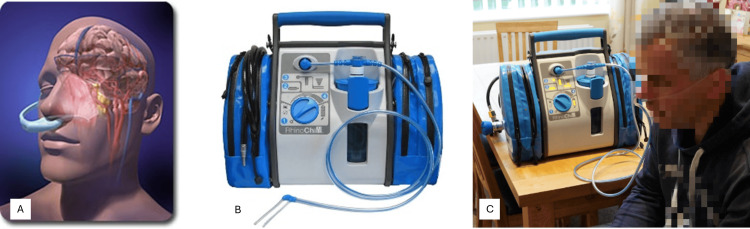
RhinoChill® intranasal cooling system. (A) Schematic, (B) picture of the control unit and intranasal catheter, and (C) connected device in use. The images are provided by BrainCool AB.

The RhinoChill® intranasal cooling device, which is produced by the medical device company BrainCool AB, consists of a control unit, a silicone intranasal catheter, and a one-liter bottle of coolant (Figure 1). RhinoChill® as a cooling method works by spraying a mix of liquid coolant, perfluorohexane, and oxygen or air (~20 liters per minute), via nasal catheters onto the upper surface of the nasal cavity where it evaporates and absorbs heat from the tissue, thereby cooling the tissue and the innate vasculature that supplies blood to the brain.

The coolant used is perfluorohexane (PFH), a six-chain perfluorocarbon. PFH is a colorless, odorless, radiolucent liquid. PFH belongs to a class of perfluorocarbon fluids that are fully fluorinated with no functional reactive groups.

**Figure 2:**
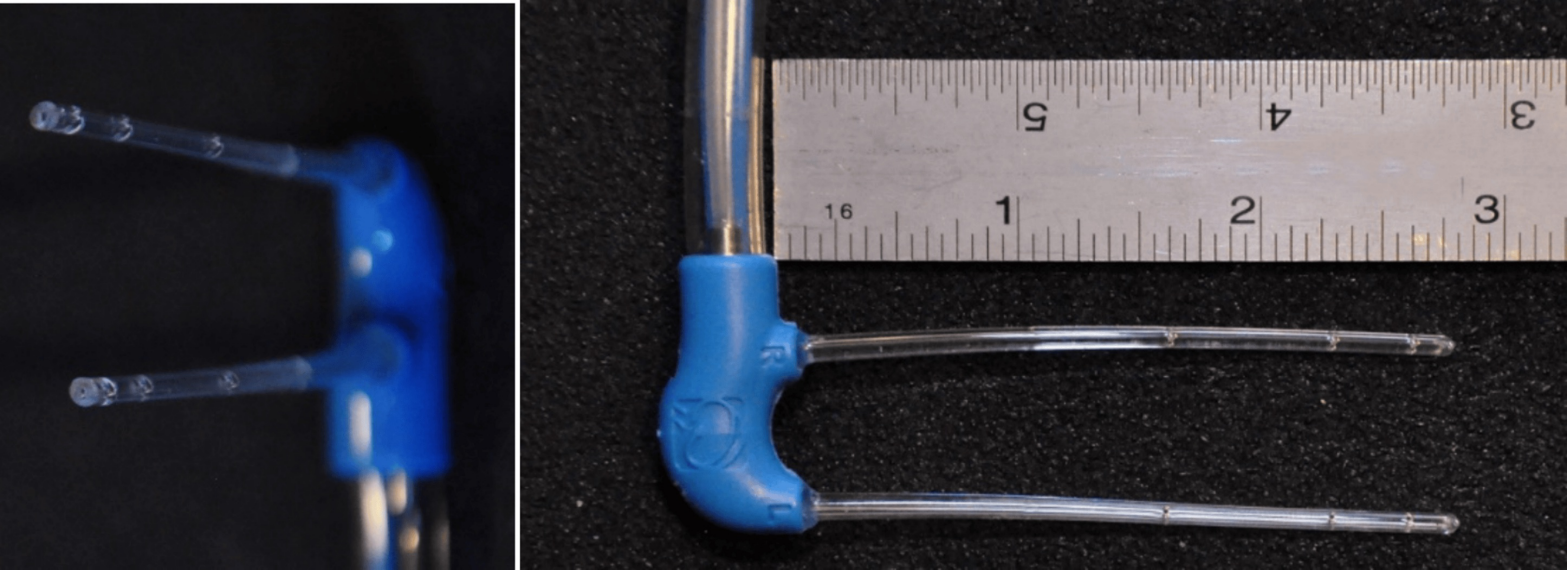
RhinoChill® intranasal migraine silicon catheter (6 centimeters) with spray ports on the upper surface.

The 6 cm long intranasal catheters have spray ports on the upper surface to distribute coolant into the nasal cavity, upwards towards the base of the skull (Figure 2). The device cools down the nasal cavity, and indirectly the circulation to parts of the brain. After each use, the nasal catheter was cleaned and disinfected to maintain its clinical cleanliness for reinsertion during subsequent treatments.

Outcome measures

The primary outcome was a reduction of migraine symptoms (headache, nausea, photophobia, and phonophobia symptoms, scored none 0, mild 1, moderate 2, severe 3) directly after treatment. We chose to evaluate migraine symptoms using a relatively simple four-point verbal rating scale to maintain high response rates, despite the extensive assessment form.

Secondary outcome measures were divided into tolerance/safety measures and effect measures.

*Tolerance/Safety Measures* 

Tolerance to RhinoChill® cooling (Visual Analogue Scale (VAS) 1-10 for pain/discomfort) and adverse events.

Effect Measures

Reduction of migraine symptoms at one, two, and 24 hours post-treatment, use of rescue migraine medication, time to freedom of pain, time to freedom of all migraine symptoms, treatment response compared to standard medication/screening period.

Statistical analysis

No power calculation was made for this study since it was not designed to detect statistically significant effects, but rather to gather preliminary data, identify potential issues, and refine the study protocols for larger clinical trials. We aimed to enroll 15 participants in the study, as this was the same number included in the RhinoChill® outpatient pilot study conducted in 2015 [16].

## Results

**Figure 3:**
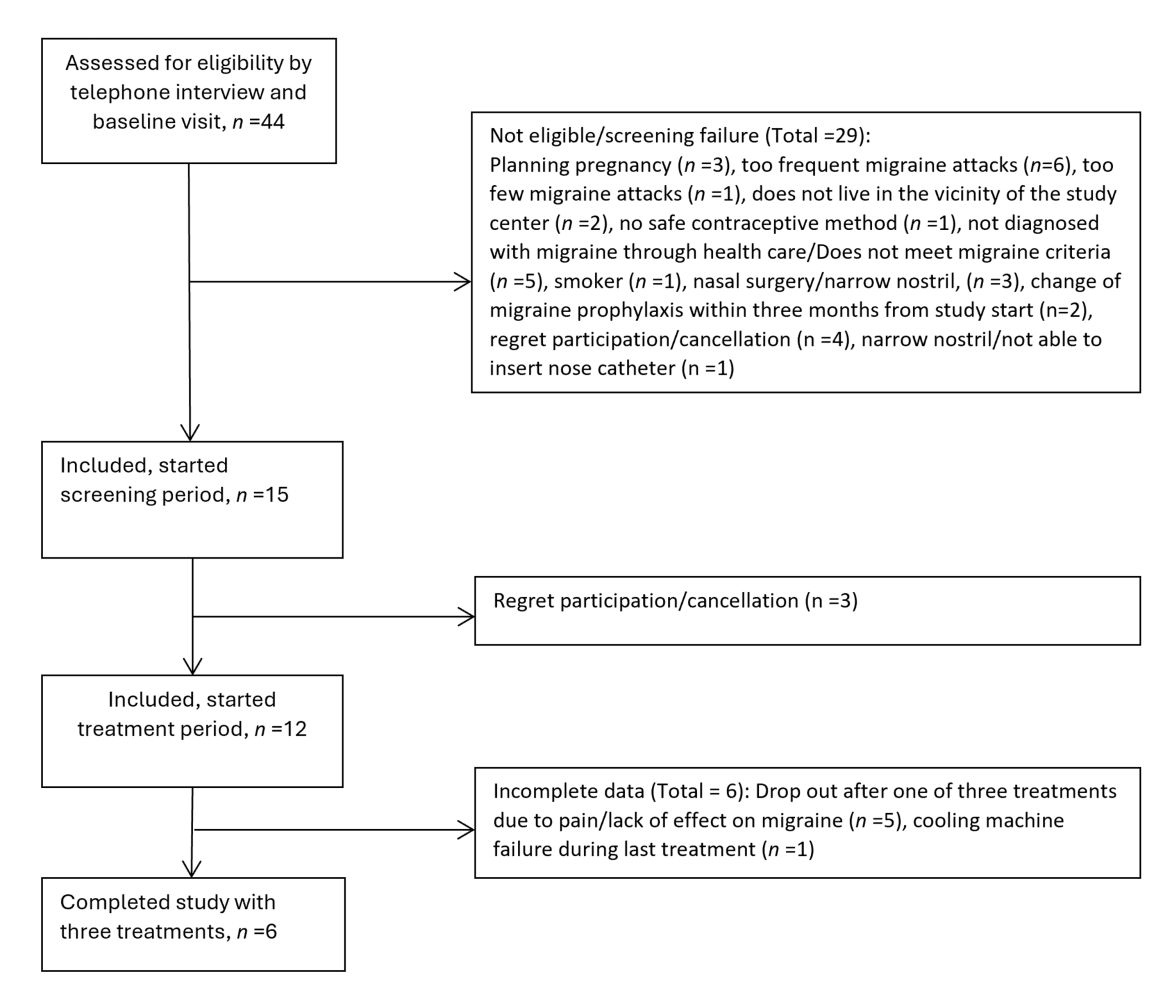
Recruitment flowchart.

Between August 2020 and April 2021, a total of 44 individuals were assessed for eligibility by telephone interview and at the baseline visit (Figure 3). Due to non-eligibility, a total of 29 were subsequently excluded. Of the 15 participants who signed the informed consent form, 12 completed screening (March-May 2021) and started the treatment period (May-November 2021). Five individuals (42%) dropped out after the first treatment due to pain/discomfort from treatment and lack of effect on migraine. Six participants completed the study with three cooling treatments.

The baseline characteristics of the 15 included participants and of those seven who fulfilled at least two cooling treatments are shown in Table 1. The mean age was 41 years and the participants had, on average, 4.5 migraine attacks per month. The proportion of women was 80%. All participants used triptans as an acute migraine treatment, and 60% also used either paracetamol or non-steroidal anti-inflammatory drugs (NSAIDs). Around 25% used prophylactic migraine treatment. There were no significant differences between those who were included and the group that completed at least 2/3 cooling treatments (Table 1).

**Table 1:** Baseline characteristics of included participants and of participants who completed ≥ 2 treatments. NSAID, non-steroidal anti-inflammatory drug; ASA, acetylsalicylic acid; CGRP, calcitonin gene-related peptide.

	Included (n=15)	Completed ≥ 2 treatments (n=7)
Demographics		
-Age in years, mean (min-max)	41 (20-62)	35 (20-52)
-Female	12 (80%)	6 (86%)
-BMI (kg/m^2^), mean	24.2	23.5
-Years since migraine diagnosis, mean (min-max)	20 (4-50)	19 (4-33)
-Average number of migraine attacks/month	4.5	5.1
-Migraine with aura	5 (33%)	1 (14%)
Active acute migraine medications		
-Triptans	15 (100%)	7 (100%)
-Paracetamol	2 (13%)	2 (29%)
-NSAID/ASA	8 (53%)	4 (57%)
Prophylactic migraine medications		
-Prophylactic treatment, ≥1 medications	4 (27%)	2 (29%)
-Tricyclic antidepressant, amitriptyline	1 (7%)	1 (14%)
-Angiotensin II receptor blocker, candesartan	3 (20%)	1 (14%)
-CGRP antagonist, erenumab	2 (13%)	1 (14%)

A total of 26 treatments were performed by 12 participants, of which 23 treatments were reported according to the clinical research form by 10 participants. The remaining three treatments were not reported because the subject chose to terminate their participation in the study (n = 2) and due to incomplete treatment caused by device failure (n = 1).

Headache intensity was reported on a four-graded scale (0 = none, 1 = mild, 2 = moderate, 3 = severe). Immediately after 10 minutes of cooling treatment, the headache intensity decreased from 1.9 to 1.3, as compared to a decrease in headache from 2.2 to 2.0 during the first 10 minutes of the screening period (Table 2). Nausea decreased by 0.2 points, photophobia by 0.2 points, and phonophobia by 0.3 points after 10 minutes of RhinoChill® treatment, which was in line with the results from the screening period.

**Table 2:** Registered migraine symptoms; comparison between screening period (treatment as usual) and treatment period (cooling treatment). 0 = none, 1 = mild, 2 = moderate, 3 = severe. NSAID, non-steroidal anti-inflammatory drug; ASA, acetylsalicylic acid.

	Screening period (n=13) Mean ± SD	Treatment period (≥1 treatment) (n=12) Mean ± SD
Pain (headache)		
-Baseline (start of registration/time of medication)	2.2 ± 0.7	1.9 ± 0.6
-10 minutes	2.0 ± 0.7	1.3 ± 1.0
-1 hour	1.6 ± 0.5	1.6 ± 0.8
-2 hours	1.0 ± 0.8	1.4 ± 0.7
-24 hours	0.4 ± 0.5	0.1 ± 0.2
Nausea		
-Baseline (start of registration/time of medication)	0.9 ± 1.0	1.0 ± 0.8
-10 minutes	0.5 ± 0.7	0.8 ± 0.6
-1 hour	0.6 ± 0.6	0.8 ± 0.8
-2 hours	0.4 ± 0.7	0.7 ± 0.9
-24 hours	0.2 ± 0.3	0.0 ± 0.1
Photophobia		
-Baseline (start of registration/time of medication)	1.3 ± 0.7	1.4 ± 0.6
-10 minutes	1.2 ± 0.9	1.2 ± 0.6
-1 hour	0.8 ± 0.4	1.1 ± 0.5
-2 hours	0.3 ± 0.4	0.9 ± 0.5
-24 hours	0.2 ± 0.4	0.0 ± 0.1
Phonophobia		
-Baseline (start of registration/time of medication)	1.2 ± 0.8	1.1 ± 0.7
-10 minutes	1.0 ± 0.8	0.8 ± 0.8
-1 hour	0.6 ± 0.7	0.8 ± 0.7
-2 hours	0.3 ± 0.4	0.7 ± 0.6
-24 hours	0.2 ± 0.3	0.0 ± 0.0
Time to freedom of pain (hours)	12.0 ± 10.0	13.0 ± 13.6
Time to freedom of all migraine symptoms (hours)	25.3 ± 16.9	16.0 ± 13.6
Acute migraine medication		
-Number of participants taking ≥1 migraine medication	13 (100%)	
-Triptans	7.5 (58%)	
-Paracetamol	0.5 (4%)	
-NSAID/ASA	5 (38%)	
Repeated medication during migraine attack	5 (38%)	
Use of acute rescue medication		
-Proportion of participants taking rescue medication		60%
-Proportion triptans of medication taken		65%
-Proportion paracetamol of medication taken		6%
-Proportion NSAID/ASA of medication taken		29%
Time to rescue medication (hours)		5.5 (5.8)

During 60% of the cooling treatments participants took rescue medication, on average 5.5 hours after migraine onset. Time to freedom of pain was similar between the cooling and screening (13 and 12 hours, respectively). Time to freedom of all migraine symptoms was shorter during cooling treatment compared to screening (16 and 25 hours, respectively). There were no serious adverse events reported during the study, but several side effects/non-serious adverse events (Table 3).

**Table 3:** Symptoms before and after treatment, percent (times reported/total number of treatment sessions) unless stated otherwise.

	Before treatment	After treatment
Sinus pressure	13% (3/23)	35% (8/23)
Blocked nose	4% (1/23)	39% (9/23)
Runny nose	0	43% (10/23)
Nosebleed	0	22% (5/23)
Nose discomfort	0	61% (14/23)
Dry eyes	4% (1/23)	4% (1/23)
Time until symptoms ceased after treatment (hours)		13.5 (14.8)

The side effects were self-limiting and non-serious in nature, but bothersome and in some cases long-lasting. The most reported side effects were nose discomfort (61%) and runny nose (43%). In approximately one-fifth of the treatment sessions, the participants experienced nosebleeds. 

On a VAS from 0 - 10 (no discomfort - unbearable) the cooling treatment experience was rated 7.0 on average by those completing two or more treatments, and 7.4 on average by all participants completing at least one treatment.

**Figure 4:**
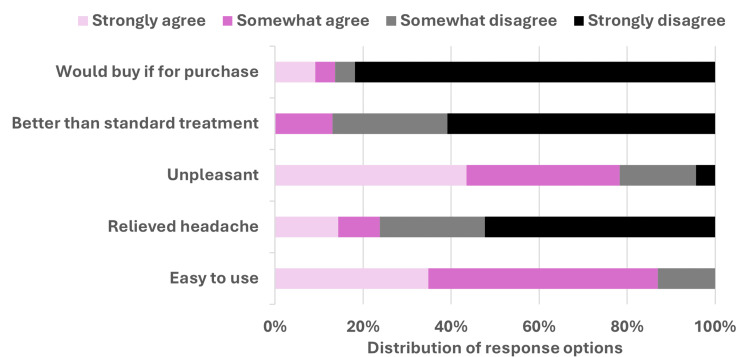
Functionality of device. Feedback from 11 participants after 23 treatment sessions (one to three treatments/person).

One device error was reported, where the device turned off eight minutes into treatment. The device was rated as being easy to use in the feedback, but the treatment itself as unpleasant, and with insufficient effect on headaches. If the device was available for purchase, only one study participant thought it was better than their current acute treatment and stated they would use it for their migraine. The other 11 participants who tried the device stated they would not buy it (Figure 4). Recurring comments in free text concerned pain during cooling treatment and discomfort due to running coolant from the nose and throat.

## Discussion

We found that self-administered intranasal cooling with the RhinoChill® device at home in acute migraine attacks may have small short-term effects on headache, and that time to freedom of all migraine symptoms may decrease. However, the treatment was considered unpleasant, painful, and less effective than regular acute migraine medication, thus leading to a large drop-out rate. The results of this study, showing the non-feasibility of the intranasal cooling intervention with the RhinoChill® device at home, led to the cancellation of the planned RCT.

Since a large proportion of people with migraine experience insufficient effects from currently available medications [18,19], the use of complementary and alternative medicine for treating migraine is widely accepted and applied [20,21]. It is important to evaluate the effect and safety of alternative treatments, highlight good examples, and advise against ineffective or harmful ones. To our knowledge, this is the first study to evaluate intranasal cooling treatment in an at-home setting.

We acknowledge that a major limitation of this study is the small sample size and lack of a control group. We aimed to get a sample size of 10-15 participants to be able to conduct this pilot study. The large drop-out rate after the first administered treatment left a small number of treatments. However, the number of completed treatments was greater in our study than in the previous RhinoChill® outpatient clinic study. Despite a large dropout rate, we managed to get feedback from all participants who tried at least one treatment and were thus able to establish that the main reason for the dropout was pain and a perceived lack of effect from the treatment. The recruitment method used in the study is likely to target individuals who are seeking or open to novel treatment methods, and thus may not be representative of a standardized population. This could introduce biases in both efficacy and treatment adherence. Likely, a population without this selection bias would have had less tolerance for the discomfort associated with the cooling treatment.

A strength of the study is that the screening period registrations serve as controls for the comparison of effect between cooling treatment and usual care.

The previous RhinoChill® pilot study showed a statistically significant reduction of pain and associated symptoms after treatment of acute migraine attacks in the hospital outpatient clinic with the RhinoChill® device [16]. The participants in the in-hospital pilot study from 2015 did not report the same degree of discomfort as the participants in this study, possibly because of the out-patient clinic setting and the fact that treatment delivered by healthcare professionals gives the patient a better sense of safety than when they must self-administer it alone in their home [16]. Another explanation as to the different study results could be that the silicone catheter was reduced in length from 10-6 cm for our study, which could have led to reduced cooling and thus a reduced effect on pain. On the other hand, this change was made because the previous study showed an effect regardless of how far the catheter was inserted into the nose. The larger catheter would also have been harder to handle in the at-home setting.

Our study showed that the time to freedom from all migraine symptoms may decrease with cooling compared to usual treatment. However, these results are difficult to evaluate because a majority of the participants took rescue medication in addition to cooling.

Although our study results were discouraging to some extent and led to the cancellation of a planned large-scale RCT with the current RhinoChill® device, there are several intervention studies that have shown promising results from cooling treatment for acute migraine [7]. However, many of these studies have been small and of questionable power to determine clinically relevant effects. Larger studies are needed given the high number of individuals suffering from migraine and the high number of those not responding to triptans or having contraindications for the use of triptans. If nasal cavity cooling were to be introduced as a new kind of acute migraine treatment it would have to be further developed, so that the discomfort reported during treatment was minimized, and the effect on migraine pain and associated symptoms stronger than what was seen in this pilot study.

## Conclusions

RhinoChill® intranasal cooling treatment in an at-home setting was found to be unpleasant and had only small effects on migraine pain. The results from this pilot study were intended to further develop and improve the intervention and study design prior to the implementation of a large-scale multicenter RCT in Sweden and Norway. However, the results instead led to the cancellation of the planned large-scale RCT.

It is important that we continue exploring and developing new non-pharmacological treatments for persons with migraine. Further development of more user-friendly and less cumbersome methods of delivering cold therapy can be one such goal.

## References

[1] Steiner TJ, Stovner LJ, Jensen R, Uluduz D, Katsarava Z (2020). Migraine remains second among the world's causes of disability, and first among young women: findings from GBD2019. J Headache Pain.

[2] Vos T, Abajobir AA, Abate KH (2017). Global, regional, and national incidence, prevalence, and years lived with disability for 328 diseases and injuries for 195 countries, 1990-2016: a systematic analysis for the Global Burden of Disease Study 2016. Lancet.

[3] Ashina M, Katsarava Z, Do TP (2021). Migraine: epidemiology and systems of care. Lancet.

[4] Stovner LJ, Andree C (2010). Prevalence of headache in Europe: a review for the Eurolight project. J Headache Pain.

[5] Arnott J (1849). Practical illustrations of the treatment of the principal varieties of headache by the local application of benumbing cold; with remarks on the remedial and anaesthetic uses of congelation in diseases of the skin and surgical operations. London: J. Churchill 1849.

[6] Zanchin G, Maggioni F, Granella F, Rossi P, Falco L, Manzoni GC (2001). Self-administered pain-relieving manoeuvres in primary headaches. Cephalalgia.

[7] Hsu YY, Chen CJ, Wu SH, Chen KH (2023). Cold intervention for relieving migraine symptoms: a systematic review and meta-analysis. J Clin Nurs.

[8] Friedman MH, Peterson SJ, Behar CF, Zaidi Z (2001). Intraoral chilling versus oral sumatriptan for acute migraine. Heart Dis.

[9] Landy SH, Griffin B (2000). Pressure, heat, and cold help relieve headache pain. Arch Fam Med.

[10] Sprouse-Blum AS, Gabriel AK, Brown JP, Yee MH (2013). Randomized controlled trial: targeted neck cooling in the treatment of the migraine patient. Hawaii J Med Public Health.

[11] Ucler S, Coskun O, Inan LE, Kanatli Y (2006). Cold therapy in migraine patients: open-label, non-controlled, pilot study. Evid Based Complement Alternat Med.

[12] Goadsby PJ, Holland PR, Martins-Oliveira M, Hoffmann J, Schankin C, Akerman S (2017). Pathophysiology of migraine: a disorder of sensory processing. Physiol Rev.

[13] Ho TW, Edvinsson L, Goadsby PJ (2010). CGRP and its receptors provide new insights into migraine pathophysiology. Nat Rev Neurol.

[14] Benemei S, Dussor G (2019). TRP channels and migraine: recent developments and new therapeutic opportunities. Pharmaceuticals (Basel).

[15] Numazaki M, Tominaga M (2004). Nociception and TRP channels. Curr Drug Targets CNS Neurol Disord.

[16] Vanderpol J, Bishop B, Matharu M, Glencorse M (2015). Therapeutic effect of intranasal evaporative cooling in patients with migraine: a pilot study. J Headache Pain.

[17] (2004). The International Classification of Headache Disorders: 2nd edition. Cephalalgia.

[18] Thorlund K, Sun-Edelstein C, Druyts E (2016). Risk of medication overuse headache across classes of treatments for acute migraine. J Headache Pain.

[19] Manack AN, Buse DC, Lipton RB (2011). Chronic migraine: epidemiology and disease burden. Curr Pain Headache Rep.

[20] Adams J, Barbery G, Lui CW (2013). Complementary and alternative medicine use for headache and migraine: a critical review of the literature. Headache.

[21] Rossi P, Di Lorenzo G, Malpezzi MG, Faroni J, Cesarino F, Di Lorenzo C, Nappi G (2005). Prevalence, pattern and predictors of use of complementary and alternative medicine (CAM) in migraine patients attending a headache clinic in Italy. Cephalalgia.

